# Supplementary material for: No effect of attentional modulation by spatial cueing in a masked numerical priming paradigm using continuous flash suppression (CFS)
Source: PeerJ. 2023 Jan 6;11:e14607. doi: 10.7717/peerj.14607 (PMC9828280; doi:10.7717/peerj.14607)
Supplement: Supplemental Information 1 [file peerj-11-14607-s001.docx]

# No effect of attentional modulation by spatial cueing in a masked numerical priming paradigm using continuous flash suppression (CFS)

Handschack, Juliane; Rothkirch, Marcus, Sterzer, Philipp; Hesselmann, Guido

**Supplementary Information**


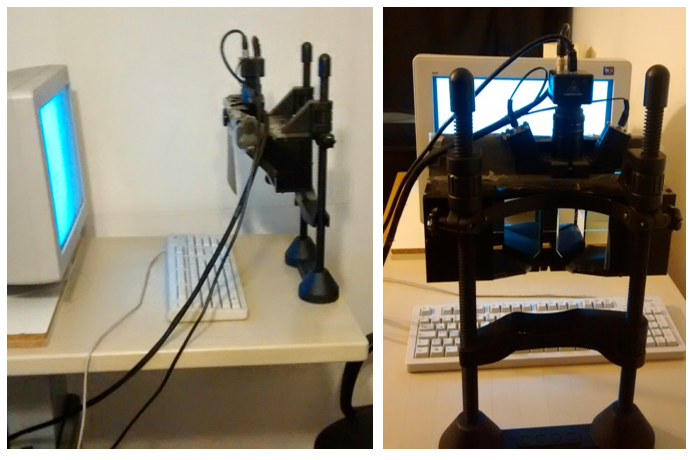


**Figure S1.** Setup. Mirror stereoscope used for dichoptic stimulation, mounted eye tracker (Cambridge Research Systems, UK), chin rest, CRT screen, and PC keyboard on a height-adjustable table. Photographs courtesy of Apoorva Madipakkam.


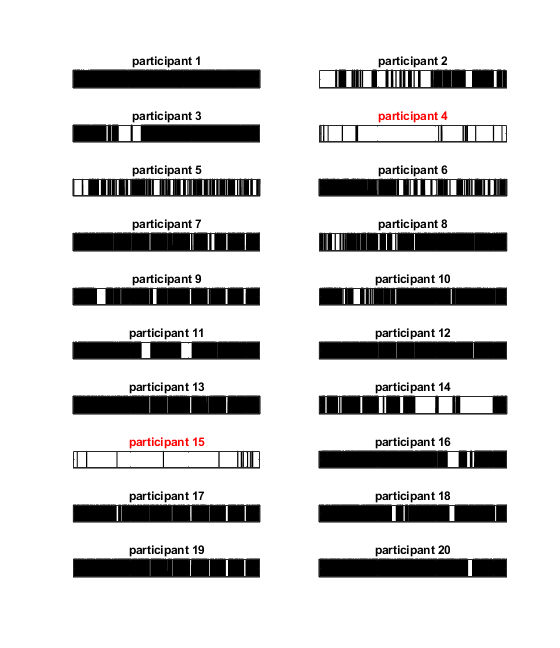


**Figure S2.** Eye-tracking data (N=20). Each horizontal bar illustrates the timeline of the main experiment and shows the available data for each participant during stimulus presentation. Black stripes correspond to registered gaze data, white areas represent missing data. Two participants were excluded due to insufficient recorded data (1.46%, 0.84%, marked in red). Please note that the numbers (1-20) above each data bar correspond to the consecutive numbers of available eye-tracking data sets, not to participants’ IDs used in the main text.


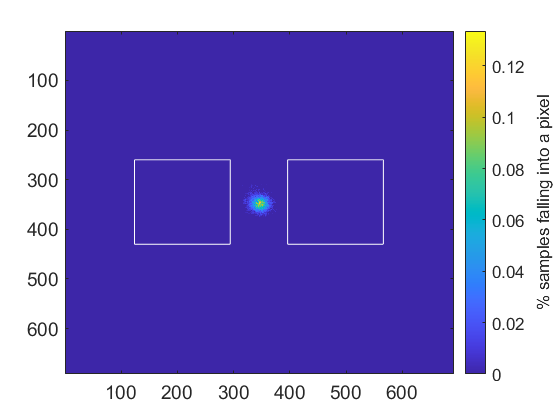


**Figure S3**. Heatmap of eye-tracking data (N=18). The heatmap shows the spatial distribution of eye gaze during stimulus presentation intervals. Brighter colours depict a larger amount of gaze data falling into a pixel. The left and right white framed rectangles represent the two boxes in which the digit and letter stimulus were presented. Note that the participants’ left eye was tracked, and that this figure shows the left side of the screen.


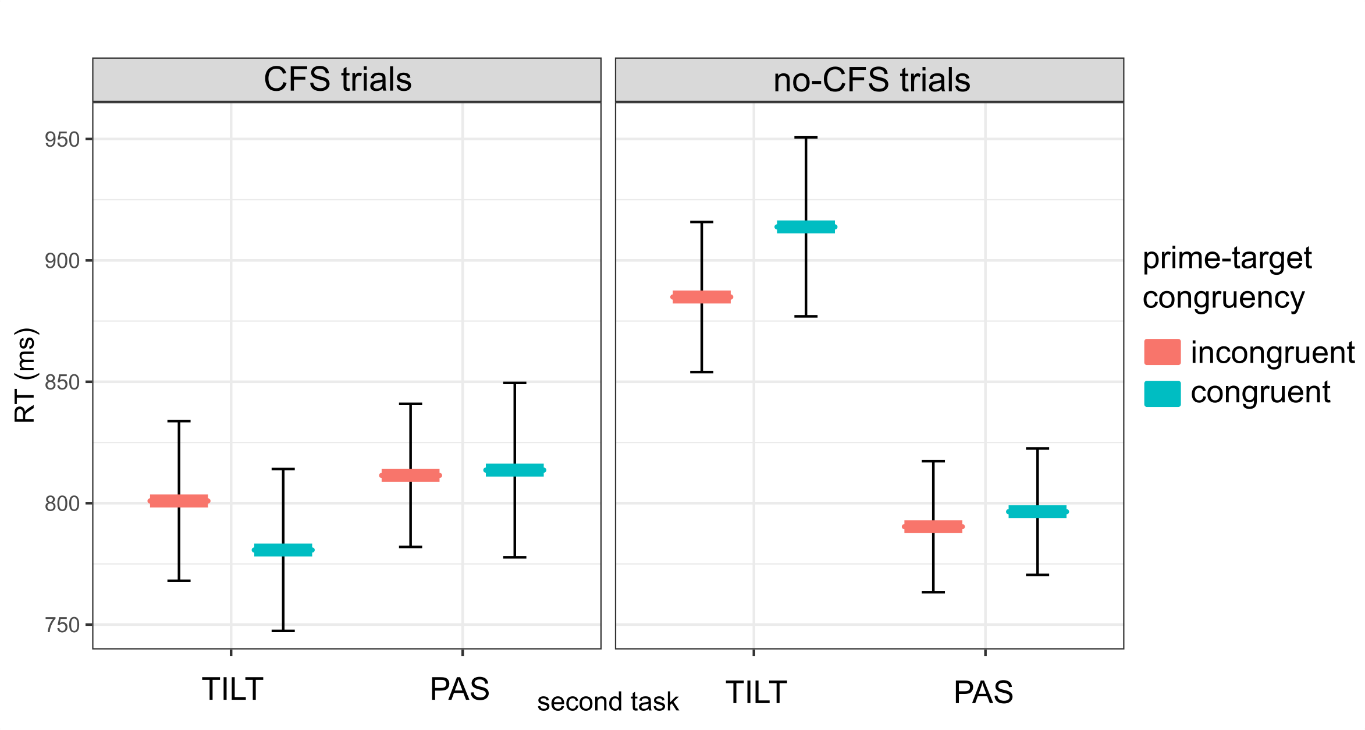


**Figure S4.** Mean RTs (N=25) from the “compare the target number to five” task in the main experiment. RTs in CFS trials (“prime invisible”, left panel) and no-CFS trials (“prime visible”, right panel), separately for prime-target incongruent trials (red) and prime-target congruent trials (blue). “TILT” refers to trials in which the second task required participants to decide whether the attended stimulus was tilted or not; “PAS” refers to trials in which participants provided visibility ratings of the attended stimulus, using the perceptual awareness scale (PAS). Error bars indicate 95% confidence intervals calculated for within-subject data using the summarySEwithin function from the Rmisc package (version 1.5.1).
